# Anal protrusion of an ileo-colic intussusception in an adult with persistent ascending and descending mesocolons: a case report

**DOI:** 10.1186/1756-0500-6-42

**Published:** 2013-02-02

**Authors:** Peter A Ongom, Robert L Lukande, Josephat Jombwe

**Affiliations:** 1 Colorectal Surgery Unit, Department of Surgery, School of Medicine, Makerere College of Health Sciences, Makerere University, P O Box 7072, Kampala, Uganda; 2 Histopathology Unit, Department of Pathology, School of Biomedical Sciences, Makerere College of Health Sciences, Makerere University, P O Box 7072, Kampala, Uganda; 3 Colorectal Surgery Unit, Department of Surgery, Mulago Hospital, P O Box 7051, Kampala, Uganda

**Keywords:** Ileo-colic intussusception, Anal protrusion, Persistent mesocolon, Azygosis, Zygosis

## Abstract

**Background:**

Intussusception is one of the less common causes of intestinal obstruction among adults. It is usually covert (concealed) in its clinical presentation. The ileo-colic type with accompanying anal protrusion is extremely rare. The case at hand is that of both an ileo-colic intussusception with anal protrusion, in the presence of a persistence of both the ascending and descending mesocolons; a case possibly yet to be documented in literature.

**Case presentation:**

A 32 year-old African-Ugandan woman presented with complaints of a mass protruding per anus for 2 weeks. It was reducible and associated with colicky abdominal pain, loose stools, and bloody-mucoid discharge per anus. She had previously had a one and a half month’s history of abdominal pain; periodically continuous, while other times colicky in character. Examination and investigations revealed an intussusception with a partial intestinal obstruction. At laparotomy she was found to have an ileo-colic intussusception with a freely mobile colon throughout its length. There were persistent ascending and descending mesocolons, and absent hepatocolic and splenocolic ligaments. The intussusceptum was ‘milked’ but not completely reducible. A right hemicolectomy was done, with ileo-transverse colonic anastomosis. Histopathological examination revealed no preexisting pathologic lesion as a lead point.

**Conclusion:**

The persistence of the ascending and descending mesocolons (azygosis) best explains the anal protrusion of an ileo-colic intussusception with partial obstruction. In this case zygosis (normal retroperitoneal ascending and descending colonic positioning) failed embryologically. This experience is particularly beneficial to general surgeons, radiologists, gastroenterologists, colorectal surgeons and pathologists.

## Background

Adult intussusception accounts for less than 5 per cent of intussusceptions with an incidence of 2 to 3 per 1,000,000 of the population per year [1]. This translates to 1 to 3 per cent of intestinal obstruction cases. It is the invagination of a segment of intestine into another, usually in a proximal-to-distal direction. The invaginated part of gut is referred to as the intussusceptum, while the portion into which it invaginates is the intussuscepiens. The classic triad of symptoms: cramping, vomiting, and rectal bleeding are not as obvious in adults as among children, thus making it difficult to diagnose with an even greater delay before treatment. However, as opposed to children, 90% of adult intussusceptions are associated with an identifiable etiology. This etiologic factor is best described as a lead point; a pathological or structural/anatomical lesion at the apex of the intussusceptum [2]. About two-thirds of these lead points are malignant tumors, with less than one-third resulting from benign processes. Hence, there is definitely a need to identify the underlying causes of adult intussusception and provide the necessary definitive treatment.

The protrusion (also referred to as prolapse) of the intussusceptum through the anal verge is rare, especially in adults. There are a number of documented cases in the literature, each tending to have some uniqueness about it [3,4]. Even with this in perspective, children tend to be more affected with up to 29 per cent having a prolapse with intussusception [5]. Ileo-sigmoid intussusception with anal prolapse in an adult has been reported [6]. It was associated with ischaemia and necrosis.

During embryologic development the mesenteries of the ascending and descending parts of the colon blend with the posterior abdominal wall peritoneum by the process of zygosis [7]. This constitutes the final process of intestinal development after all other rotations of gut. Failure of completion of this leaves a persistent right mesocolon in about 25 per cent of the population, and a left one in about 33 per cent [8]. Clinical complications that have been associated with these anatomical variations include: primary intestinal obstruction [9], and colonic volvulus as a result of a persistent descending mesocolon [10]. Persistence of the mesocolon of both, ascending and descending parts of the colon, has manifested as colonic varices [11].

## Case presentation

A 32 year-old African-Ugandan woman of nilo-hamitic ethnicity presented to her local hospital with complaints of abdominal pain and bloody diarrhea for 5 days in mid-August, 2012. She was admitted there and treated for amoebic dysentery, though no stool examination had been done. The abdominal pain was generalized and lasted for long periods prior to subsiding. Stools were mucoid and foul-smelling, though not copious in amount. She reportedly improved following empiric treatment with metronidazole, cotrimoxazole, analgesics and IV fluids, and generally felt better for a period of about 2 weeks. Subsequently, her abdominal symptoms changed in character. She felt an abdominal mass and the abdominal pain became colicky in nature, and was associated with vomiting. There was straining while defecating, tenesmus and a feeling of incomplete voiding of stool. A few days later she noticed what she described as "something protruded from the anus". It came as a result of straining to pass stool, and spontaneously reduced. Over the next one week she found that she had to manually reduce it, occasionally. She was consequently referred with a working diagnosis of rectal prolapse.

The patient presented to our hospital on the 23rd September, 2012 with a chief complaint of abdominal pain and “a mass protruding from the anus”. She also complained of a swelling in the abdomen. The pain was colicky and she still passed bloody-mucoid stools, interspersed occasionally with semi-solid stools. This she did with straining and only passed very small quantities. She vomited once or twice daily, through not profusely, and passed flatus. Remarkable physical examination findings were per abdomen and per rectum. She had a firm, mobile mass in the umbilical region that measured 14 cm by 11 cm; located intra-abdominal and only slightly tender. The rest of the abdomen was of normal fullness. On rectal examination, the anal verge was normal. No protruding mass was visible (it had spontaneously reduced). A mobile mass was palpable 3 cm from the verge, and was free of the rectal mucosa all round. The examining digit could not get to its proximal limit; characteristic of intussusception. There was plenty of mucoid discharge and some dark blood. Significantly elsewhere, she was mildly dehydrated and afebrile. She had a blood pressure of 130/80 mmHg and a pulse rate of 80 beats/min. Examination of the mucoid, blood tinged material was negative for ova and cysts of any parasite.

**Figure 1:**
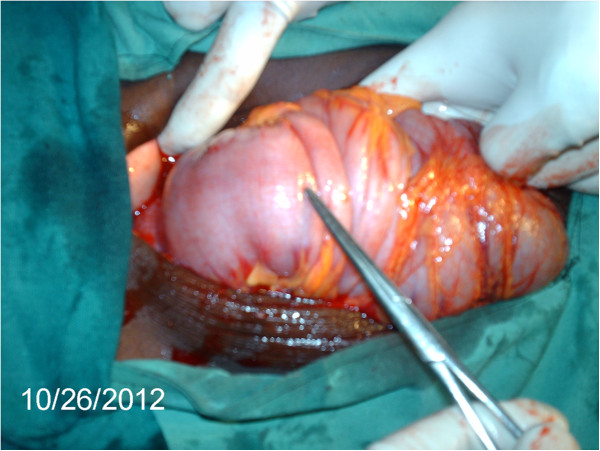
**‘Mass’ containing intussusception; umbilical region. **The photograph shows the intussusception encountered upon opening the anterior abdominal wall, just deep to the umbilical region, through a midline incision. This had been palpated as an abdominal mass during the patient’s examination. Virtually the whole colon is invaginated and ‘crammed’ within this mass of gut, occupying a relatively small volume of space.

A diagnosis of a prolapsed intussusception with partial intestinal obstruction was made. An abdominal ultrasound scan confirmed this. Our patient was resuscitated and prepared for an exploratory laparotomy. This was performed through a midline incision. On opening the peritoneum, a large section of gut was revealed, ‘crammed’ and invaginated, but stretching from the distal ileum right up to the sigmoid colon, occupying the central aspect of the abdominal cavity (Figure 1). This caused a partial intestinal obstruction. There was no ascites and no distended bowel loops proximal to this. This mass of gut constituted an intussusception with the intussusceptum stretching from the ileum, through the entire colon, right to the rectum, with intermittent anal protrusion (prolapse).

**Figure 2:**
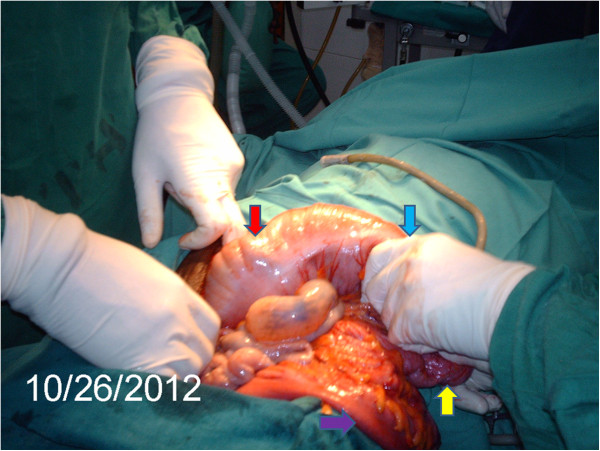
**Manual reduction of the intussusception. **Manual reduction (‘milking’) of the intussusceptum being done in a distal to proximal direction (light blue arrow). Distally, the now empty sigmoid colon (purple arrow) and descending colon (yellow arrow) are illustrated. More proximally shown is the proximal part of the transverse colon continuous with the ‘free’ ascending colon (red arrow). Note the freedom of the entire colon and the apparent absence of the hepatocolic ligament and hepatic flexure of the colon.

**Figure 3:**
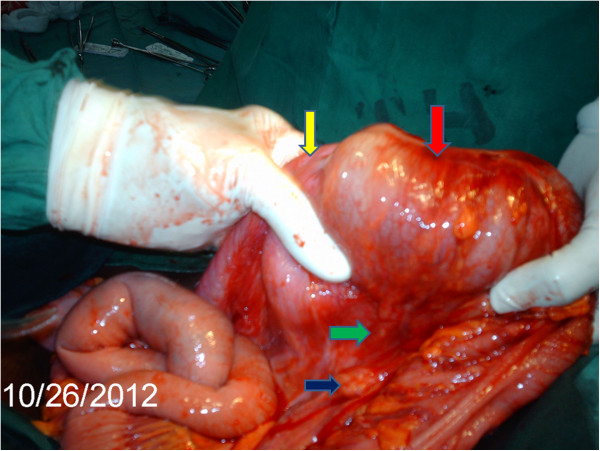
**Intussusception following maximal manual reduction, requiring resection. **The photograph shows the intussusception after ‘milking’ it as much as possible, yet still incompletely reduced. Yellow arrow – ileum at the point of invagination into the caecum/ascending colon. The ileal mesentery is grossly thickened. Red arrow – thickened intussusceptum and intussuscepiens at the ascending colon. Green arrow – illustrates the persistent ascending mesocolon. Deep blue arrow – enlarged lymph node of the mesentery. Histopathological examination showed reactive hyperplasia.

**Figure 4:**
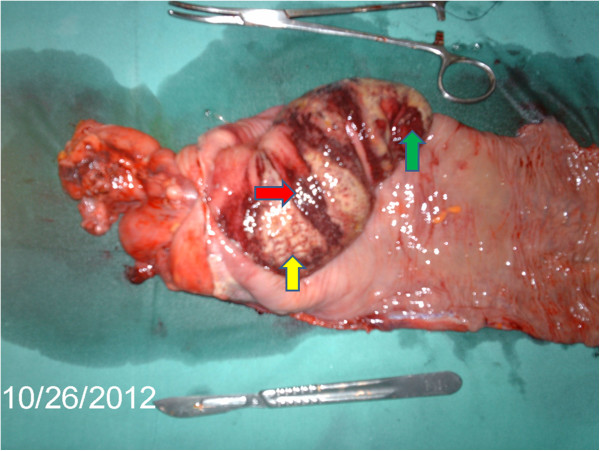
**Dissection of the resected intussusception. **Green arrow – lead point of the intussusceptum (ileocaecal junction). Yellow arrow – ischaemic changes over the intussusceptum. Red arrow – haemorrhagic areas. Note the relative size of the irreducible intussusceptum in comparison with the surgical instruments (scalpel and artery forceps).

**Figure 5:**
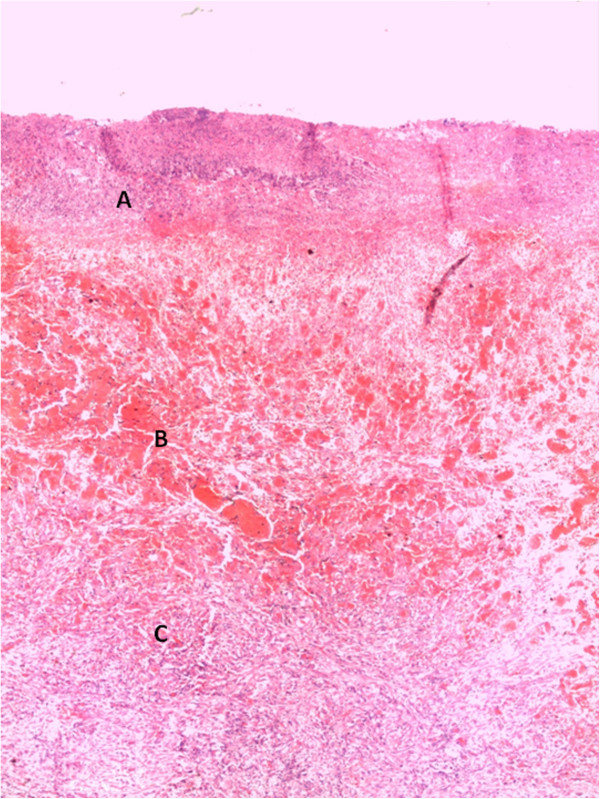
**Hematoxylin & Eosin stained section of colon. **Part **A **shows totally necrotic mucosa with a necro-inflammatory infiltrate. Part **B **shows submucosa with intense vascular congestion. Part **C **shows muscularis propria with features of fibrosis.

The intussusception was then manipulated. Its intussusceptum’s tip was felt in the sigmoid colon upon tagging on it. The intussusceptum was gently ‘milked’ proximally (Figure 2) but could not be freed entirely. The most conspicuous findings were absent hepatocolic and splenocolic ligaments, and the presence of prominent ascending and descending mesocolons. These allowed for free mobility of the colon from is most proximal to distal extents; no part was fixed, retroperitoneally. The mesentery of the right colon had enlarged, soft and discrete lymph nodes and was oedematous (Figure 3). Complete reduction of the intussusception by manual reduction was not possible at the proximal portion (ileo-cecal junction, cecal and proximal ascending colon region). A right hemicolectomy was done with primary ileo-transverse colon, end-to-side anastomosis. All other abdominal and pelvic organs were essentially normal. No intraperitoneal adhesions were found. The anterior abdominal wall was closed in layers. The resected segment was opened revealing thickening of the ileo-cecal junction and adjacent areas, with necrotic mucosa overlying the intussusceptum (Figure 4). However, the mucosa was regular with no polyps, ulcers or constrictions; no gross picture of neoplasia (Figure 4) or pre-existing benign structural pathologic lesion. The resected specimen was submitted for histopathological examination. This showed necrotic mucosa and fibrosis of the muscularis propria (Figure 5). The lymph nodes showed reactive follicular hyperplasia. There were neither features of amoebiasis nor malignancy. The patient's post-operative course was uneventful.

## Discussion

This case presents a scenario of chronicity of intussusception with partial intestinal obstruction. The symptoms found in adult patients with intussusception are often chronic and non-specific, such as abdominal pain, fever, nausea, vomiting, melena stools, weight loss, and constipation [2]. Physical examination may demonstrate diffuse or localized abdominal tenderness, while an abdominal mass is detected in a minority of patients, about 24 per cent to 42 per cent of cases [12,13]. We recognize a number of these features in our patient.

An interesting aspect of this case is that she was initially clinically diagnosed with amoebic dysentery, a common condition in the tropics, presenting with abdominal pain (occasionally crampy), bloody-mucoid stools, diarrhea and vomiting. Amoebic dysentery is thus a top differential diagnosis in this patient. In chronic amoebic infestation with a resultant amoebic granuloma, intussusception may result as well. Relevant literature on this is expressed in a Sri Lankan study [14] where about 50% of intussusceptions occurred in adults (a rather unusual incidence), and had a maximum age incidence in the fourth decade. The clinical picture of intussusception was characteristic, and a mass was palpable in 90% of the patients, facilitating diagnosis without ancillary investigations. On the basis of the histological examination of resected specimens it was concluded that amoebic granulomatous formation in the dependent ‘diverticulum’ of the caecum was the predisposing cause of the caecocolic intussusception, accounting for the chronicity of a large number of cases. Irrespective of the duration of the illness, gangrene did not occur in any of the cases of this type, although resection was occasionally required on account of irreducibility. In view of the proliferation of fibrous tissue in the wall of the cecum, complete evagination of the intussusception could only be achieved by surgical exploration and manipulation. This picture mirrors our case. However in our case, amoebiasis and granuloma formation were ruled out at histopathological examination.

Anal protrusion of the intussusceptum should not present a conundrum in the descriptive diagnosis of this case. She was referred with a new diagnosis of rectal prolapse, another differential diagnosis of intussusception. The two can usually be easily distinguished. In the event of a rectal prolapse, there is palpable continuity between the perianal/anal tissue and the protruding tissue. In contrast, in intussusception no palpable continuity may be felt; typically manifested in this case. Though ultrasonography (very specific for intussusception diagnosis) was done, it was not essential. The clinical features are sufficient to reach a definitive diagnosis of intussusception.

The ability of the ileo-cecal lead point to ‘easily’ reach the anal verge and prolapse through is plausibly explained by the freedom of the colon made by the persistence of ascending and descending mesocolons. This is our hypothesis. Following normal embryonic zygosis, ascending and descending colons are firmly held in position (retroperitoneal) and would not allow for a relatively smooth invagination of an intususceptum all the way from the terminal ileum to the rectum and out. Right from the initiation of the invagination of the intussusceptum with its accompanying luminal and vascular obstruction, a vicious cycle will be offset. This will consist of: oedema, obstruction, and ischaemia; with subsequent accentuation of each of these with time. The result is more likely to be an intussusceptum with its lead point stopping within the ascending or transverse colon, and associated with total luminal obstruction. However, with failure of zygosis, as in our case, there is persistence of ascending and descending mesocolons, with more room for manouvreability of the intussusceptum. Further still, this mobile colon with ‘free’ hepatic and splenic flexures allows for only a partial intestinal obstruction with no gross abdominal distension, and the ability to pass flatus and stool, albeit still bloody and mucoid.

Previous reports advocate for reduction of adult intussusception instead of resection [15] especially if the course is idiopathic following radiologic investigations (computerized tomography). However, chronic intussusception does not always allow for a successful manual reduction to be performed, due to thickening, fibrosis and cross-scarring within the intussusceptum. We also have the paramount concern of possible malignancy [1], with approximately 65% of lead points being due to benign or malignant neoplasms [13]. The reduction of an intussusception especially when small bowel is significantly involved is advantageous in preserving a considerable length of bowel. In our case, we reduced it manually till it could not be ‘milked’ smoothly, anymore. We then chose the option of a right hemicolectomy with the view that if it had been a malignancy we would have achieved definitive surgical treatment, yet concurrently preserving more ileum and distal transverse colon. The option of resection without any reduction, in the absence of gangrene, did not apply in our case. We essentially had the entire colon involved, and would have had to do a pancolectomy with a longer ileal resection.

## Conclusion

Our case, to the best of our knowledge, is the first documented one of its kind, at least in the East African region. It is an unusual presentation of a known, though rare, clinical disease (ileo-colo-rectal intussusception with anal protrusion) coexisting with a rare anatomical variation (persistent ascending and descending mesocolons). We draw lessons from the clinical features which generally give a mixed picture. There were both typical (crampy abdominal pain, bloody/mucoid stools and vomiting) and atypical features (little or no abdominal distension, and the presence of a diffuse umbilical mass). Firstly, a mobile, abdominal mass in the umbilical region, with a protruding intussusception yet only a partial intestinal obstruction, should raise the suspicion of persistent mesocolons. Secondly, persistent mesocolons allow for only a partial intestinal obstruction even with such a sizeable intussusception in place.

Clinicians ought to have a high index of suspicion for intussusception even when treating for amoebic dysentery. Either of the two can occur alone or contemporaneously. It is important to do stool analysis prior to initiation of antiparasitic/antibiotic agents so that we can ascertain or rule out amoebiasis. Radiological investigations (Ultrasound scan especially) are important for early definite intussusception diagnosis.

## Consent

Written informed consent was obtained from the patient for publication of this Case Report and any accompanying images. A copy of the written consent is available for review by the Editor-in-Chief of this journal.

## Competing interests

The authors declare that they have no competing interests.

## Authors’ contributions

OAP managed our patient peri-operatively, performed the operation, and wrote the manuscript. LRL prepared and analyzed the resected specimen and histopathological slides. He also edited the manuscript. JJ participated in the management of our patient and edited the manuscript. All authors read and approved the final manuscript.
